# Comment on: Longitudinal risk of serious infections in patients with inflammatory arthritis on immunomodulating therapy compared to controls: Reply

**DOI:** 10.1093/rap/rkaf069

**Published:** 2025-06-05

**Authors:** Ingrid Egeland Christensen, Siri Lillegraven, Till Uhlig, Sella Aarrestad Provan

**Affiliations:** Center for Treatment of Rheumatic and Musculoskeletal Diseases (REMEDY), Diakonhjemmet Hospital, Oslo, Norway; Center for Treatment of Rheumatic and Musculoskeletal Diseases (REMEDY), Diakonhjemmet Hospital, Oslo, Norway; Center for Treatment of Rheumatic and Musculoskeletal Diseases (REMEDY), Diakonhjemmet Hospital, Oslo, Norway; Institute of Clinical Medicine, Faculty of Medicine, University of Oslo, Oslo, Norway; Center for Treatment of Rheumatic and Musculoskeletal Diseases (REMEDY), Diakonhjemmet Hospital, Oslo, Norway; Public Health Section, Inland Norway University of Applied Sciences, Elverum, Norway

Dear Editor, We thank Rebecca Parry for taking an interest in our study and taking the time to respond to our manuscript [1].

Parry raises important questions that are relevant for clinical practice. Due to the low number of events, we did not have the statistical power to break down the infection rates across each specific drug. Although we do have data on the type of diagnosis, there is limited generalizability due to few events. Analyses of the type of infection and exploration of possible trends in the type of serious infection across time periods of treatment initiation with a biologic or targeted synthetic disease-modifying antirheumatic drug (DMARD) would require an even larger sample size than at hand from the NOR-DMARD study.

Parry states that there are data suggesting stable rates of infection for patients treated with rituximab and refers to a study assessing the long-term safety of rituximab in patients with rheumatoid arthritis [2]. However, this study does not compare the risk of serious infection with rituximab to the other biologic DMARD therapies. Rituximab has been found to have an increased risk of serious infections compared with other biologics in large observational registry studies [3, 4], supporting our statement regarding rituximab in the limitation section.

Finally, we agree with Parry that the risk of serious infections during treatment with immunomodulating drugs is an important issue that needs awareness during follow-up of these patients. Future studies are needed to better understand the impact of other risk factors for infection apart from immunomodulating therapy, such as comorbidities and concomitant glucocorticoid treatment, to optimize clinical care according to the individual risk of serious infection during immunomodulating therapy.

## Data Availability

No new data were analysed or generated in support of this article.
